# Estimated protection against COVID-19 based on predicted neutralisation titres from multiple antibody measurements in a longitudinal cohort, France, April 2020 to November 2021

**DOI:** 10.2807/1560-7917.ES.2023.28.25.2200681

**Published:** 2023-06-22

**Authors:** Tom Woudenberg, Laurie Pinaud, Laura Garcia, Laura Tondeur, Stéphane Pelleau, Alix De Thoisy, Françoise Donnadieu, Marija Backovic, Mikaël Attia, Nathanael Hozé, Cécile Duru, Aymar Davy Koffi, Sandrine Castelain, Marie-Noelle Ungeheuer, Sandrine Fernandes Pellerin, Delphine Planas, Timothée Bruel, Simon Cauchemez, Olivier Schwartz, Arnaud Fontanet, Michael White

**Affiliations:** 1 Infectious Disease Epidemiology and Analytics G5 Unit, Department of Global Health, Institut Pasteur, Université Paris-Cité, Paris, France; 2 Emerging Diseases Epidemiology Unit, Institut Pasteur, Université Paris-Cité, Paris, France; 3 Structural Virology Unit, Department of Virology and CNRS UMR 3569, Institut Pasteur, Université Paris-Cité, Paris, France; 4 Molecular Genetics of RNA Viruses, Department of Virology, Institut Pasteur, Université Paris-Cité, CNRS UMR 3569, Paris, France; 5 Mathematical Modelling of Infectious Diseases Unit, Institut Pasteur, Université Paris-Cité, UMR2000, CNRS, Paris, France; 6 Hôpital de Crépy-en-Valois, Crépy-en-Valois, France; 7 Laboratoire de virologie, CHU Amiens, AGIR UR4294, UPJV, Amiens, France; 8 Clinical Investigation and Access to Research Bioresources (ICAReB) platform, Center for Translational Science, Institut Pasteur, Paris, France; 9 Center for Translational Science, Institut Pasteur, Paris, France; 10 Virus and Immunity Unit, Department of Virology, Institut Pasteur, Université Paris-Cité, Paris, France; 11 PACRI Unit, Conservatoire National des Arts et Métiers, Paris, France

**Keywords:** SARS-CoV-2, neutralising antibodies, SARS-CoV-2, COVID-19, seroprevalence, humoral immunity, protection, antibodies, neutralising antibodies, viral immunity, sero-epidemiology

## Abstract

**Background:**

The risk of SARS-CoV-2 (re-)infection remains present given waning of vaccine-induced and infection-acquired immunity, and ongoing circulation of new variants.

**Aim:**

To develop a method that predicts virus neutralisation and disease protection based on variant-specific antibody measurements to SARS-CoV-2 antigens.

**Methods:**

To correlate antibody and neutralisation titres, we collected 304 serum samples from individuals with either vaccine-induced or infection-acquired SARS-CoV-2 immunity. Using the association between antibody and neutralisation titres, we developed a prediction model for SARS-CoV-2-specific neutralisation titres. From predicted neutralising titres, we inferred protection estimates to symptomatic and severe COVID-19 using previously described relationships between neutralisation titres and protection estimates. We estimated population immunity in a French longitudinal cohort of 905 individuals followed from April 2020 to November 2021.

**Results:**

We demonstrated a strong correlation between anti-SARS-CoV-2 antibodies measured using a low cost high-throughput assay and antibody response capacity to neutralise live virus. Participants with a single vaccination or immunity caused by infection were especially vulnerable to symptomatic or severe COVID-19. While the median reduced risk of COVID-19 from Delta variant infection in participants with three vaccinations was 96% (IQR: 94–98), median reduced risk among participants with infection-acquired immunity was only 42% (IQR: 22–66).

**Conclusion:**

Our results are consistent with data from vaccine effectiveness studies, indicating the robustness of our approach. Our multiplex serological assay can be readily adapted to study new variants and provides a framework for development of an assay that would include protection estimates.

**Key public health message**

**What did you want to address in this study?**

We wanted to measure the protection against COVID-19 in the population, after much build-up of immunity following transmission of SARS-CoV-2 and vaccinations. Measuring SARS-CoV-2-specific antibodies in blood samples can indicate previous infection and vaccination coverage in a population. To date, population-wide antibody testing has not been able to provide quantitative estimates of protection against COVID-19.

**What have we learnt from this study?**

We measured antibody levels to SARS-CoV-2 in 905 individuals, and used these to predict how protective these antibodies are against COVID-19. How protective these antibodies were translated into estimates of population-level immunity. Protection was better with more vaccinations.

**What are the implications of your findings for public health?**

Our novel method allowed us to go beyond seroprevalence estimates and enabled us to assess the protection against COVID-19 in a large population sample. Using our serological laboratory test with variant-specific antibodies, we showed that we were able to identify under-protected individuals who may be targeted with additional vaccine doses.

## Introduction

The COVID-19 pandemic has resulted in substantial morbidity and mortality worldwide, with over 6.8 million deaths and over 760 million confirmed cases reported up to March 2023 [1]. In addition to infection-acquired immunity, a large share of the world’s population has been vaccinated against the causative pathogen, severe acute respiratory syndrome coronavirus 2 (SARS-CoV-2). By March 2023, 85% of adults 18 years and older in European Union/European Economic Area (EU/EEA) countries had received at least one dose of vaccine [2].

Population-level immunity can be measured with serology-based assays. Seroprevalence is determined by measuring the presence of antibodies to a particular protein of SARS-CoV-2, typically the whole spike protein, a smaller component of the spike protein such as its receptor-binding domain (RBD) or S2 subunit, or the nucleocapsid protein (NP). The presence of anti-spike antibodies is consistent with immunity as a result of immunisation, whereas the presence of anti-NP antibodies indicates previous infection. While the presence of antibodies is associated with protection against infection, it is not always predictive of protection against COVID-19 [3]. Unlike most serological assays, a neutralisation assay measures antibodies that can block viral replication and infection of cells. These so-called neutralising antibodies are more likely to provide protection as they possess true antiviral activity. Despite strong individual correlations between antibody levels and neutralisation activity, individuals with similar IgG levels following vaccination were regularly observed to have substantially varying neutralisation titres [4].

From clinical trials examining neutralisation titres and efficacy estimates from COVID-19 vaccine trials, it was shown that neutralisation titres correlate very well with protection against symptomatic infection and hospitalisation [5-8], with higher neutralisation titres associated with higher vaccine efficacy. With the emergence of new SARS-CoV-2 variants that partly escape immunity, neutralisation titres decreased considerably. Neutralisation titres of SARS-CoV-2 Delta (Phylogenetic Assignment of Named Global Outbreak (Pango) lineage designation B.1.617.2) and Alpha (Pango lineage designation B.1.1.7) variants were observed to be fourfold and 1.6-fold less, respectively, compared to the neutralisation titres of ancestral strains [9,10]. For these variants, the variant-specific neutralisation titres remained strongly correlated with protection against symptomatic infection with SARS-CoV-2 [11]. However, Omicron BA.1 caused a further reduction in neutralisation. Relative to the Delta variant, neutralisation titres of sera with high antibody levels reduced 6–23-fold against Omicron BA.1 [12].

Assessment of population-level immunity can provide critical information in the response to the SARS-CoV-2 pandemic, e.g. by identifying vulnerable subgroups in need of control measures such as booster vaccine doses. Neutralisation titres are not optimal for population-level surveillance for several reasons, including the time needed to process large sample numbers and the required cell-culture equipment that is not present in many laboratories. Widespread measurement of population-level immunity requires high-throughput assays that can be more easily implemented in diagnostic laboratories. Here, we aimed to correlate the measurement of variant-specific antibodies with neutralisation titres, from which we inferred the protection against symptomatic and severe COVID-19.

## Methods

### Study design

**Figure 1:**
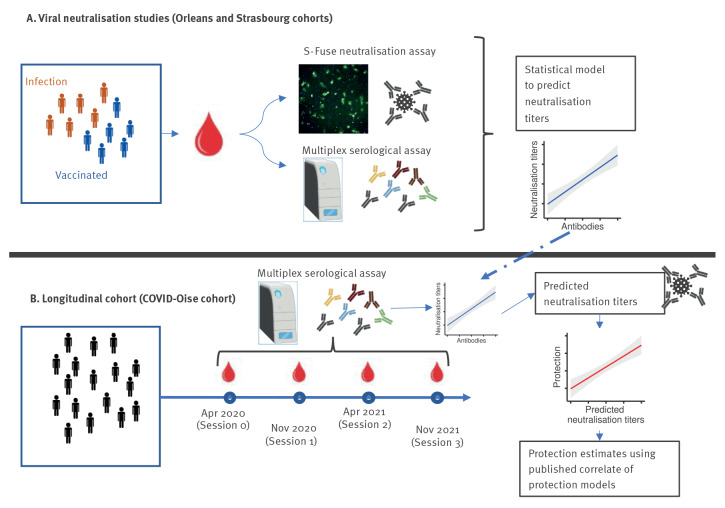
Overview of the study design, France, April 2020–November 2021

We developed a multiplex serological assay to measure the binding of antibodies of different isotypes to a variety of SARS-CoV-2 antigens, as well as the strength of these interactions. The correlation between these measurements and neutralisation titres against multiple SARS-CoV-2 variants was used to develop a prediction model for serum samples. From the predicted neutralisation titres, we used previously developed models [5,11] to translate these titres into individual-level protection estimates. We applied this method to a longitudinal cohort study of ca 900 individuals followed for up to 20 months, between April 2020 and November 2021 (Figure 1).

A. Establishment of a prediction model for neutralisation activity based on measured antibody levels using data from viral neutralisation studies comprising both infected (Orleans and Strasbourg cohorts) and vaccinated (Orleans cohort) individuals [9,10].

B. The longitudinal cohort (COVID-Oise cohort) was followed up to 20 months with four sampling occasions (Sessions 0, 1, 2 and 3). Serum samples were taken and analysed for antibody levels. These antibody levels were used to predict neutralisation titres, which were used to estimate protection estimates.

### Samples

#### Viral neutralisation studies

To correlate antibody measurements with neutralisation titres, we collected 304 serum samples from individuals with either vaccine-induced or infection-acquired immunity to SARS-CoV-2. These individuals were enrolled in two different clinical cohort studies (i.e. Orleans and Strasbourg cohorts), described elsewhere in more detail [9,10]. Individuals who participated in the Orleans cohort were either convalescent or vaccinated. In this study, researchers aimed to describe the persistence of specific and neutralisation antibodies over a 24-month period starting in August 2020. Convalescent serum was sampled 6 or 12 months after infection. Vaccinations occurred in the first quarter of 2021. Sampling started from 2 weeks after the first dose and continued until 6 months after the second dose. The second clinical cohort study, the Strasbourg cohort, included convalescent individuals only and was initiated in April 2020. The serological status of these individuals was assessed at 3 or 6 months after symptom onset.

#### Longitudinal cohort

One of the first clusters of COVID-19 in France was detected in the town of Crépy-en-Valois in the Oise Department. In winter 2020, scientists at Institut Pasteur initiated a longitudinal cohort study, named the COVID-Oise cohort. Participants comprised a wide age range (5–101 years), including children up to nursing home residents. The inclusion criteria were to live, work and/or study in the area of the city of Crépy-en-Valois (Oise, France, ca. 15,000 inhabitants) at the time of study initiation. No exclusion criteria were applied. Participants were invited four times for collection of epidemiological data and serum samples. Data and samples from sessions held in November 2020 (Session 1), April 2021 (Session 2) and November (Session 3) were used in this analysis. Many of the COVID-Oise participants also participated in earlier studies that took place in April 2020 (Session 0) [13]. Collected data and biological specimens from these earlier studies were integrated to the analyses in the current manuscript.

### Serological assays

Samples were tested with three serological assays, described in more detail in the Supplementary Methods. First, we used a bead-based multiplex serological assay (Luminex) to measure immunoglobulin G (IgG) and immunoglobulin A (IgA) antibodies against up to 30 antigens [14], as well as avidity [15]. This assay included the spike and RBD of several SARS-CoV-2 variants, namely the ancestral strain (Wuhan lineage with substitution D614G), Alpha, Beta (Pango lineage designation B.1.351) and Delta. The median fluorescence intensity (MFI) produced by the MAGPIX system was used for analysis. A five-parameter logistic curve was used to convert MFI to relative antibody units (RAU), relative to the standard curve (a two-serial dilution from 1:50 to 1:102,400) performed on the same plate to account for inter-assay variation. The second assay was a Luciferase-Linked ImmunoSorbent Assay (LuLISA). The LuLISA was used as a validation for the Luminex assay and for the determination of seropositivity measuring antibodies to SARS-CoV-2 spike ectodomain, and NP. The third assay was the S-Fuse neutralisation assay. This assay measures to what extent S-Fuse cells become infected with SARS-CoV-2 in the presence of samples of sera of the participants. S-Fuse cells exhibit a bright green fluorescence upon syncytia formation. The effective dilution 50% (ED50) was calculated with a reconstructed curve using the percentage of the neutralisation at the different concentrations. Viral stocks of different SARS-CoV-2 variants were produced on Vero E6 cells, titrated on Vero E6 or S-Fuse cells and sequenced to confirm viral lineages (data not shown).

### Statistical analyses

Relative antibody units, neutralisation and protection estimates were visualised by vaccination and infection status. Vaccination status was self-reported. Infection status was determined through either a positive SARS-CoV-2 PCR result or serology. Date of infection status was determined with the following strategy: (i) a positive PCR or antigen test confirmed by positive serology, (ii) clinical diagnosis of COVID-19 by a medical doctor before the first positive serology, (iii) starting date of at least one self-reported symptom (fever, cough, dyspnoea, agueusia/dysgueusia or anosmia/dysnomia) before a positive serology or (iv) circulation of SARS-CoV-2 within a household or nursing home before a positive serology. If no infection date could be determined based on these four strategies, we used the mid-point between last negative serology and first positive serology. If no negative serology was available, the mid-point was calculated between 1 January 2020 and first positive serology.

Serological classification of previous infection was dependent on vaccine status. For unvaccinated participants, we developed a random forest algorithm based on RAU. This algorithm was trained on samples from both PCR-confirmed cases of COVID-19 and negative control samples, and calibrated to have 99% specificity [16]. For vaccinated individuals, positivity was based on antibody levels to NP with both the LuLISA and the Luminex assay.

Protection in this study is defined to be against symptomatic and severe COVID-19. These two terms have been used in similar ways in the various efficacy studies used by Khoury et al. [5]. Symptomatic COVID-19 was defined as a positive SARS-CoV-2 PCR result combined with at least one typical symptom, such as fever, cough, shortness of breath, chills, new or increased muscle pain, agueusia/dysgueusia, anosmia, sore throat, diarrhoea, or vomiting. Severe COVID-19 was defined as confirmed COVID-19 with any of the following additional features: respiratory failure, evidence of shock, significant acute renal, hepatic, or neurologic dysfunction, admission to an intensive care unit or death.

#### Estimation of neutralisation titres

To establish a model to predict neutralisation titres with RAU from our multiplex assay, we tested samples from the viral neutralisation studies with both the Luminex assay and the S-Fuse neutralisation assay, and used the data to build random forest regression models. As we had SARS-CoV-2 variant-specific antigens for RBD and whole spike for four variants (ancestral, Alpha, Beta and Delta), we developed four random forest regression models in parallel. For each random forest, the number of trees was set at 1,000. Regressions were built in a stepwise manner. The first antigen in the regression was selected based on the importance of that antigen, measured by the mean decrease in accuracy on the out-of-bag samples. Subsequently, all other variables were added one by one to identify the most important antigen in the regression. The antigen associated with the lowest sum of residual sum of squares among the four different variant-specific random forest regression models was kept in the model. This process was repeated until no further decrease in the lowest residual sum of squares was observed.

#### Estimation of protection

Neutralisation titres were normalised by the average neutralisation activity in convalescent serum 3 weeks following symptom onset. Normalised neutralisation titres were converted into protection estimates using models developed by Khoury et al. and Cromer et al. [5,11]. In short, the relationship between in vitro neutralisation levels and the observed protection from SARS-CoV-2 infection was studied using immunogenicity data from phase 1 and 2 studies of seven vaccines and data on protection from corresponding phase 3 studies. A logistic model was used to describe the relationship. Subsequently, this model was extended with data from 24 studies on in-vitro neutralisation and clinical protection in order to incorporate the loss of neutralisation to SARS-CoV-2 variants.

## Results

### Estimation of neutralisation titres

**Figure 2:**
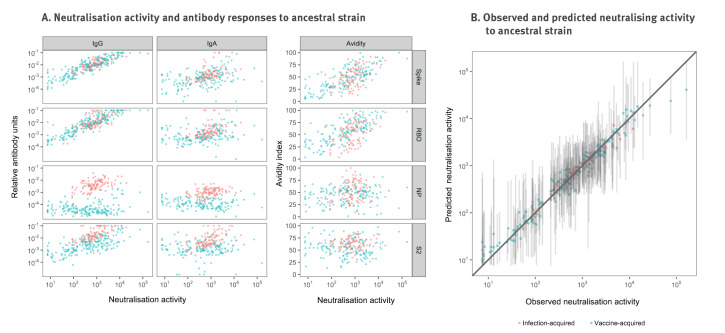
Establishment of a prediction model for SARS-CoV-2 neutralisation activity based on antibody levels from viral neutralisation studies, France, April 2020–March 2021 (n = 304 serum samples)

NP: nucleocapsid protein; RBD: receptor-binding domain; SARS-CoV-2: severe acute respiratory syndrome coronavirus 2.

For the 304 samples used for the viral neutralisation studies, we measured neutralisation titres to several SARS-CoV-2 variants (ancestral, Alpha, Beta, Delta) as well as IgG and IgA antibody levels and avidity to all SARS-CoV-2 antigens. Of these, 106 samples had immunity acquired through an infection and 198 had vaccine-acquired immunity, of whom the majority (53%) were vaccinated twice with Comirnaty (BNT162b2, BioNTech-Pfizer). The median age was 56 years (range: 42–60), and most samples were from men (n = 108, 55%). Further details on the sex, age and immunity status are provided in Supplementary Table S1. We found a strong correlation between neutralisation titres and RAU, especially antibodies targeting the spike and RBD of isotype IgG (Figure 2A). The highest correlation was observed between IgG antibodies to the spike protein (r = 0.87) and neutralisation activity. Visualisations for Delta are depicted in Supplementary Figure S1.

A. Correlation between relative antibody units of spike, RBD, NP and S2 for IgG, IgA and avidity, and neutralising activity to the ancestral SARS-CoV-2 strain.

B. Prediction of neutralisation activity to the ancestral strain of SARS-CoV-2. The predictions were derived from a random forest regression model containing the following biomarkers: spike IgG, RBD IgG, spike IgG avidity, S2 IgG, RBD IgA and RBD IgG avidity.

With random forest regression models, we found a strong association between RAU with variant-specific neutralisation titres. Observed and predicted neutralisation titres to the ancestral strain are shown in Figure 2B. The development of the random forest regression models and neutralising activity of other variants can be found in Supplementary Figure S2. The final random forest regression comprised spike IgG, RBD IgG, Spike IgG avidity, S2 IgG, RBD IgA and RBD IgG avidity.

An alternative approach to predict neutralisation titres to SARS-CoV-2 variants would be to measure neutralisation titres to the ancestral strain, and then adjust for the fold reduction in neutralisation titres between variants. Taking into account the lower limit of detection, we described the relationships between neutralising titres of the ancestral strain, and Delta and Omicron BA.1 variants using a censored linear regression model, found in Supplementary Figure S3. We found that neutralisation activity of samples against the ancestral strain decreased on average by 62% (95% CI: 57–67) against Delta. A further decrease of 97.7% (95% CI: 97.1–98.3) was observed with Omicron BA.1 compared with the ancestral strain.

### Longitudinal cohort

**Figure 3:**
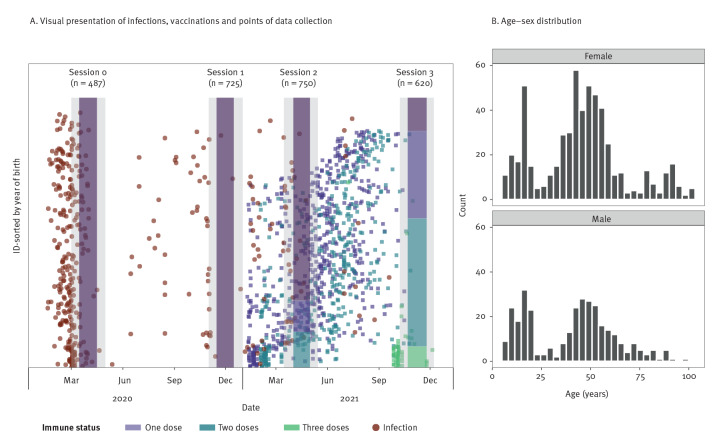
Data collection and study population of the longitudinal cohort, France, April 2020–November 2021 (n = 2,582 serum samples)

The longitudinal (COVID-Oise) cohort was established in winter 2020 (Session 1, n = 725) and two follow-up sessions took place in spring 2021 (Sessions 2, n = 750) and winter 2021 (Session 3, n = 620) (Figure 3). During these three sessions, 905 individuals were enrolled and 2,582 sera samples were collected in total. The initial studies held in spring 2020 led to the collection of a total of 487 sera samples for the participants who would later be enrolled in the COVID-Oise study. The median age of the participants was 45 years (range: 5–101) and 65% (n = 584) were female. A detailed visualisation of vaccinations, infections and participation rates can be found in Supplementary Figure S4. Among participants who participated in April 2021 (Session 2), 25% had at least received one dose of a COVID-19 vaccine. Vaccination coverage of at least one vaccine dose increased to 87% in November 2021 (Session 3).

A. Analyses were based on four sessions of data and sera collection. Vaccination coverage is shown by the four stacked bar plots. Individual vaccination dates are shown in squares, and infection dates by red circles. Vaccine doses (first, second and third dose) are indicated by coloured bars.

B. Distribution of the study population by age and sex.

**Figure 4:**
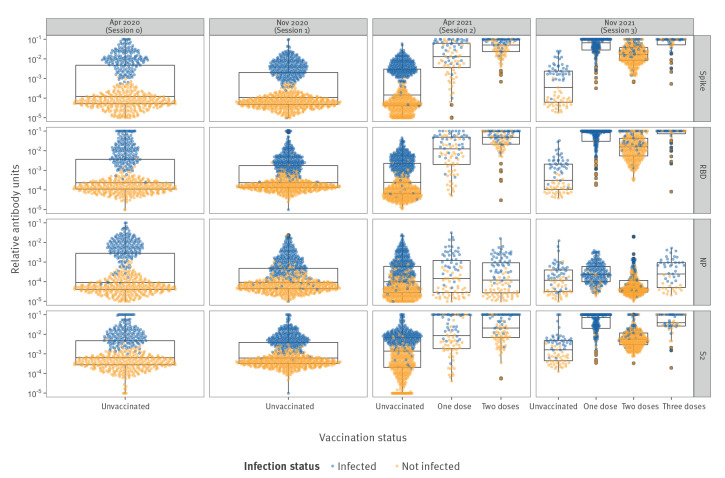
IgG antibody levels to the SARS-CoV-2 ancestral strain by vaccination status, time of sampling and type of antigen in the longitudinal cohort, France, April 2020–November 2021 (n = 2,582 serum samples)

All samples were analysed with the 30-plex serological assay, providing readouts for IgG, IgA and avidity. Among unvaccinated individuals, a clear distinction in the distribution of antibody levels to spike, RBD, NP and S2 (Figure 4) was observed. In April 2020, 36% of all samples tested positive for SARS-CoV-2 antibodies, which increased to 37% in November 2020, to 44% in April 2021 and 47% in November 2021. Antibody levels to IgA and avidity measurements by session can be found in Supplementary Figures S5–8. A comparison of the measured spike IgG and NP IgG between the Luminex multiplex assay and LuLISA showed a strong correlation, as has been observed previously [17] and examined in Supplementary Figure S9.

SARS-CoV-2: severe acute respiratory syndrome coronavirus 2.

Results are presented for participants of the longitudinal (COVID-Oise) cohort by sample collection sessions and by immune status based on vaccination (number of doses of vaccine against COVID-19) and/or past infection.

IgG antibodies are expressed as relative antibody units. Limit of the assay ranged from 0.0001 to 0.1. Colours depict infection status.

**Figure 5:**
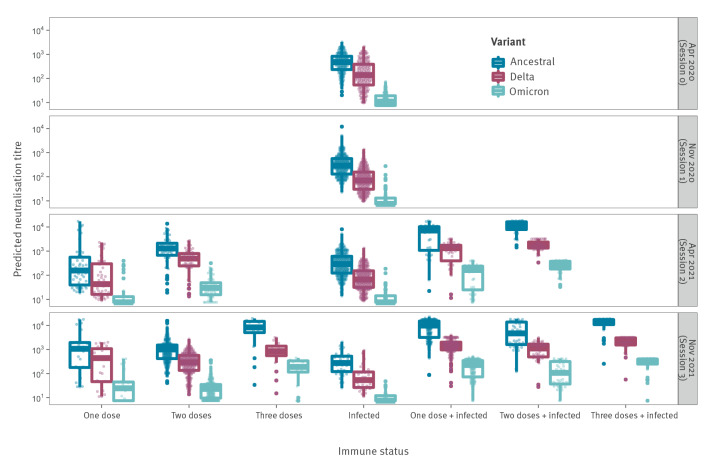
Predicted neutralisation activity to three SARS-CoV-2 variants by immune status and date of sampling in the longitudinal cohort France, April 2020–November 2021 (n = 2,582 serum samples)

Using the RAU of spike IgG, RBD IgG, S2 IgG, and RBD IgA, and the avidity index of IgG antibodies to spike and RBD, as input for our random forest regression models, we translated these measurements into variant-specific neutralisation activity. The estimated neutralisation activity to the ancestral strain, Delta and Omicron BA.1 are shown in Figure 5. Neutralisation activity to the ancestral strain and Delta were derived with random forests regression models. Neutralisation activity to Omicron BA.1 was estimated by applying the 97.7% reduction relative to neutralisation activity to the ancestral strain. 

SARS-CoV-2: severe acute respiratory syndrome coronavirus 2.

Results are presented for participants of the longitudinal (COVID-Oise) cohort by sample collection sessions and by immune status based on vaccination (number of doses of vaccine against COVID-19) and/or past infection.

Note that the neutralisation activities are only shown for individuals who had immunity because of vaccination, infection or both.

### Estimation of protection levels

**Figure 6:**
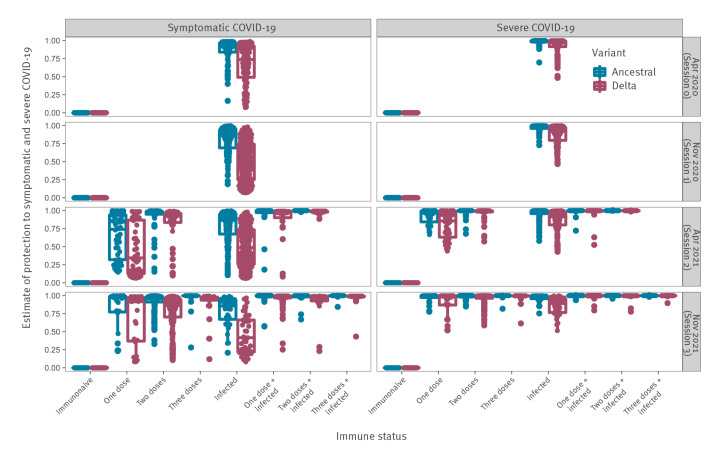
Estimates of reduced risk to symptomatic and severe COVID-19 with infection by the SARS-CoV-2 ancestral strain or Delta variant by immune status in the longitudinal cohort, France, April 2020–November 2021 (n = 2,582 serum samples)

Relative to immunonaive individuals, the risk of COVID-19 or severe COVID-19 was reduced with multiple vaccinations and/or past infection (Figure 6); the median reduced risk to COVID-19 caused by infection with the Delta variant was 42% (IQR: 22–66) among previously infected individuals and 96% (IQR: 94–98) among individuals vaccinated with three doses. In line with the reduced neutralisation titres to Delta vs the ancestral strain, protection was lower against COVID-19 and severe COVID-19 given a Delta infection. Individuals with hybrid immunity had a further reduction in risk for COVID-19 or severe COVID-19. Among individuals with vaccine-acquired immunity only, the proportion of those under-protected against symptomatic COVID-19 (defined to be a reduced risk less than 50%) with Delta infection was 35% after 1 dose, 14% after 2 doses, and 11% after 3 doses. Among individuals with hybrid immunity, the proportion of those under-protected against symptomatic COVID-19 with Delta infection was 1% after 1 dose, 5% after 2 doses, and 3% after 3 doses.

SARS-CoV-2: severe acute respiratory syndrome coronavirus 2.

Results are presented for participants of the longitudinal (COVID-Oise) cohort by sample collection sessions and by immune status based on vaccination (number of doses of vaccine against COVID-19) and/or past infection.

**Figure 7:**
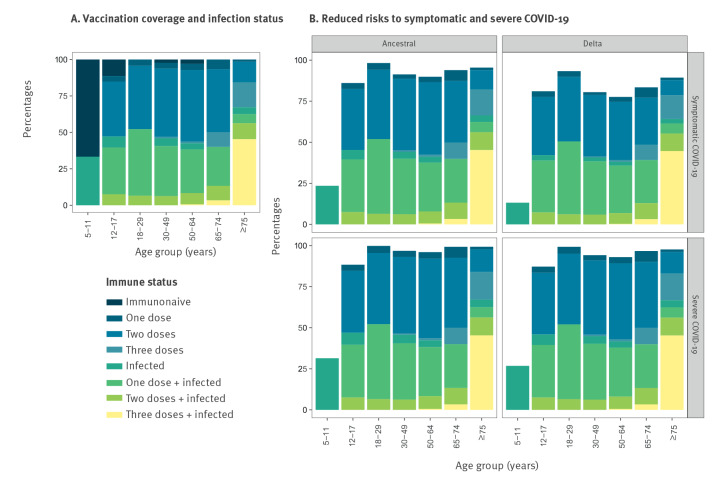
Aggregated protection against symptomatic and severe COVID-19 of participants in the longitudinal cohort, France, November 2021 (n = 620)

By aggregating individual protection estimates, we estimated the susceptibility to COVID-19 at the population level. We divided the longitudinal cohort by age group, immune status (a combination of vaccination and infection status), and summarised protection by identifying the median protection for each of these aggregated groups. The stacked protection by age group revealed that regardless of variant and severity, the groups aged 18–29 and 75 years and older had the highest reduced risk of COVID-19, partly caused by the high vaccination coverage among the oldest age group (prioritised for vaccination) and the lack of unvaccinated individuals among the 18–29-year-olds (Figure 7). For example, individuals aged 75 and older had a median reduced risk of 89% (5^th^–95^th^ percentile: 71–98) against COVID-19 with Delta variant infection and, among 18–29-year-olds, the reduced risk was 93% (5^th^–95^th^ percentile: 84–98). The 5^th^ and 95^th^ percentiles for the population-level protection for the longitudinal cohort in November 2021 can be found in Supplementary Figure S10. These findings can be extrapolated to provide an assessment of population-level immunity in the rest of France in November 2021. Based on data on vaccine doses collated by Santé Publique France and reported infections, adjusting for under-reporting [18], the immune profile of Crépy-en-Valois is representative of the rest of France. See Supplementary Figure S11 for a visualisation of the aggregated estimated protection for 13 regions in France.

A. Coverage of vaccination (number of doses of vaccine against COVID-19) and infection status by age group in participants of longitudinal (COVID-Oise) cohort in November 2021 (Session 3).

B. Stacked median reduced risks to symptomatic and severe COVID-19 by SARS-CoV-2 variant (ancestral strain and Delta variant) and age group.

## Discussion

Seroprevalence studies are widely used to provide an indication of the amount of virus transmission and vaccination coverage in a population. Immunity resulting from infection or vaccination does not guarantee prevention of infection, illness or hospitalisation by SARS-CoV-2. Our novel method allowed us to go beyond the generation of seroprevalence estimates and enabled us to determine estimates of protection. Using high-throughput multiplex assays with variant-specific antibodies, we could identify under-protected individuals who may be targeted with additional vaccine doses.

Our estimates are in line with observed vaccine effectiveness estimates for both the ancestral strain and Delta variant. Supplementary Figure S12 includes our estimated protection and vaccine effectiveness studies from observational cohort studies [19-24]. We estimated protection levels by time since vaccination, which were comparable in a series of observational and randomised controlled trials of vaccines [19-25]. We observed increased protection for individuals with hybrid immunity in line with others [26,27]. Individuals vaccinated with one dose of a COVID-19 vaccine and a confirmed infection were better protected than individuals vaccinated with two doses.

Multiple lines of evidence demonstrate that neutralising titres are associated with protection against both symptomatic and severe COVID-19 [5-8], but T-cell-mediated immunity is also known to play a critical role [28]. As many T-cell epitopes are not mutated in variants of concern, the contribution of T-cells to protective immunity is likely to remain, most notably for protection against severe COVID-19. A limitation of our study was that we assessed levels of immunity from serum only. There is clearly also a role for mucosal immunity in protecting against SARS-CoV-2 infection, especially in the case of infection-acquired immunity. During the sessions held in November 2020 (Session 1), April 2021 (Session 2), and November 2021 (Session 3), we additionally collected nasopharyngeal samples, which we plan to incorporate in future research.

Our analysis is dependent on the suitability of neutralising titres as a correlate of protection against symptomatic COVID-19, based on meta-analyses of vaccine studies [5,6]. This assumption is supported by an analysis of data from phase 3 trials of Spikevax vaccine (mRNA-1273, Moderna), which indicated that 68% of vaccine efficacy can be explained by neutralising titres [29]. This leaves up to 32% variation that may be explained by other effects such as cellular immunity or host factors. An additional limitation is that the evidence base for using neutralising titres as a correlate of protection is built on studies of infection with the ancestral strain. However, antibody levels have been observed to be associated with reduced infection with other variants, most notably Delta [30]. Although neutralising titres have frequently been shown to be associated with protection against severe COVID-19 [5-7], there is a weaker evidence base for their use as a correlate of protection. A final, important limitation to our study is that there is uncertainty in the statistical relationships used in this analysis. When considering the inferred protection from symptomatic COVID-19 obtained by analysing a sample, there will be substantial uncertainty in that individual’s estimated protection. This uncertainty will limit the use of our methods for diagnosing under-protected individuals. However, in this study we focused on aggregated protection across large numbers of samples, where the effects of uncertainty are diminished, but not removed.

Our final cross-sectional analysis of population-level protection was from November 2021. At this time, many individuals had recently received their second or third vaccine doses, and consequently had high antibody responses. In addition, many individuals had had a prior SARS-CoV-2 infection, resulting in stronger immune responses. Furthermore, there are interactions between numbers of vaccine doses and infection status, most notably as a consequence of the policy recommendation in 2021 to consider individuals with documented previous infection fully immunised after only one vaccine dose (and thereby eligible for the French ’passe sanitaire’).

The sample collection in November 2021 occurred just before the emergence of the Omicron BA.1 variant in France, replacing the previously dominant Delta variant. By accounting for the 97% reduction in neutralisation of Omicron BA.1 compared with the ancestral strain, we were able to indirectly estimate Omicron BA.1 neutralisation titres. However, we did not attempt to infer protection against Omicron BA.1 infection because of a lack of a validated correlate of protection. As a substantial proportion of the French population has been infected by the Omicron variant since November 2021, our estimates are not representative of the current immunity present in the French population.

## Conclusion

In addition to estimating seroprevalence by identifying previous infection through detection of antibodies, our approach enabled us to assess the protection against COVID-19 in a large population cohort. We were able to identify under-protected sub-groups who may be targeted with additional vaccine doses. Our multiplex serological assay and associated algorithms can be readily adapted to study new variants and provides a framework for estimating levels of protection.

## Supplementary Data

1165000 Supplement
