# Supplementary material for: Estimated protection against COVID-19 based on predicted neutralisation titres from multiple antibody measurements in a longitudinal cohort, France, April 2020 to November 2021
Source: Euro Surveill. 2023 Jun 22;28(25):2200681. doi: 10.2807/1560-7917.ES.2023.28.25.2200681 (PMC10288827; doi:10.2807/1560-7917.ES.2023.28.25.2200681)
Supplement: Supplement [file 22-00681_WOUDENBERG_SUPPLEMENT.pdf]

This supplementary material is hosted by *Eurosurveillance* as supporting information alongside the article “Protection against COVID-19 in a French longitudinal cohort from April 2020 to November 2021 based on predicted neutralization titers from multiple antibody measurements”, on behalf of the authors, who remain responsible for the accuracy and appropriateness of the content. The same standards for ethics, copyright, attributions and permissions as for the article apply. Supplements are not edited by *Eurosurveillance* and the journal is not responsible for the maintenance of any links or email addresses provided therein.

Supplementary Methods

Luminex assay

In a 96 well, non-binding microtiter plate 50 µL of protein-conjugated magnetic beads (250/region/well) and 50 µL of serum diluted 1/100 for IgG assay or 1/200 for IgA assay were mixed and incubated for 30 min at room temperature on a plate shaker. All dilutions were made in phosphate buffered saline containing 1% bovine serum albumin and 0.05% (v/v) Tween-20 (denoted as PBT). Following incubation, the magnetic beads were separated using magnetic plate separator (Luminex®) for 60 seconds and washed thrice with 100 µl PBT. The washed magnetic beads were incubated for 15 minutes with detector secondary antibody at room temperature on a plate shaker, washed thrice with 100 µl PBT and finally resuspended in 100 µL of PBT. R-Phycoerythrin-(R-PE) conjugated goat or donkey anti-human IgG antibody was used as detector antibody at 1/120 dilution and goat anti-human IgA at 1/200. A positive control pool of serum at two-fold serial dilutions from 1:50 to 1:102,400 was included on each 96 well plate. Plates were read using a Luminex® MAGPIX® system, which provides a reading of median fluorescence intensity (MFI).

A previously described 9-plex bead-based assay was extended to detect antibodies to 30 antigens in 1 µL serum or plasma samples (1). This assay allowed simultaneous detection of antibodies to 30 antigens, including stabilized trimeric Spike ectodomain (2), RBD, Membrane protein (M), Membrane Envelope protein (E), Nucleocapsid protein (NP), and a Membrane-Envelope fusion protein (ME). The trimeric Spike ectodomains and RBD antigens were produced as recombinant proteins for four SARS-CoV-2 variants, namely of the ancestral lineage, Alpha, Beta, and Delta variants. In addition, we included 8 antigens of 4 seasonal coronaviruses (Spike ectodomain and NP of NL63, 229E, HKU1, OC43). ME and Spike Sub-unit-2 (S2) SARS-CoV-2 antigens were purchased from Native Antigen (Oxford, United Kingdom) and all other antigens were produced as recombinant proteins at Institut Pasteur. The mass of proteins coupled on beads was optimized to generate a log-linear standard curve with a pool of 27 positive sera prepared from patients with reverse-transcription quantitative PCR–confirmed SARS-CoV-2 (1). We measured the levels of immunoglobulin G (IgG) and immunoglobulin A (IgA) of each sample in two separate assays. Plates were read using a Luminex MAGPIX system and the median fluorescence intensity (MFI) was used for analysis. A 5-parameter logistic curve was used to convert MFI to relative antibody units (RAU), relative to the standard curve performed on the same plate to account for inter-assay variation.

In addition to the measurement of the presence of antibodies to antigens, we also measured the strength of antibody (Ab) binding with an avidity assay .The protocol for the avidity assay was similar to the serological assay with the inclusion of an additional step. After incubation

of beads and serum samples, the complex beads-Ab were washed and then incubated for 5 minutes with 100µl of urea 6 M diluted in water, or water alone. After these 5 minutes and washing, 100µL of secondary antibodies conjugated to R-phycoerythrin (Jackson Immunoresearch) for detection of specific IgG, diluted at 1/100 was added for 15 minutes. To finish, after washing, plates were read using a Luminex® MAGPIX® system and the median fluorescence intensity (MFI) was used for analysis. The Avidity index (AI) was measured with AI = [MFI after treatment with 6M of Urea/ MFI without Urea] x 100. Avidity was only assayed for IgG.

S-Fuse neutralization assay

U2OS-ACE2 GFP1-10 or GFP 11 cells, also termed S-Fuse cells, become GFP+ when they are productively infected by SARS-CoV-2 (3, 4). Cells tested negative for mycoplasma. Cells were mixed (ratio 1:1) and plated at 8 × 103 per well in a µClear 96-well plate (Greiner Bio-One). The indicated SARS-CoV-2 strains were incubated with serially diluted monoclonal antibodies or sera for 15 min at room temperature and added to S-Fuse cells. The sera were heat-inactivated 30 min at 56 °C before use. Eighteen hours later, cells were fixed with 2% paraformaldehyde (PFA), washed and stained with Hoechst (dilution 1:1,000, Invitrogen). Images were acquired with an Opera Phenix high-content confocal microscope (PerkinElmer). The GFP area and the number of nuclei were quantified using Harmony software (PerkinElmer). The percentage of neutralization was calculated using the number of syncytia as value with the following formula: 100 × (1 − (value with serum − value in 'non-infected')/(value in 'no serum' − value in 'non-infected')). The neutralizing activity of each serum was expressed as the ED50 value. ED50 values (in µg ml−1 for monoclonal antibodies and in dilution values for sera) were calculated with a reconstructed curve using the percentage of the neutralization at the different concentrations.

Luciferase-Linked ImmunoSorbent Assay (LuLISA)

The LuLISA was used as a validation for the Luminex assay and for the determination of sero-positivity. Briefly, Nucleocapsid-specific IgG antibodies were assessed using an ELISA-based assays on sera incubated in antigen-coated wells. Antigens have been produced as follows. Full-length N protein from SARS-CoV-2 were produced with a (His)6 tag in the E. coli, purified on Ni-NTA affinity column, and then size-exclusion chromatography was performed. White 384-well plates with flat bottoms (Fluoronunc C384 Maxisorp, Nunc) were coated with 1 µg/mL of Nucleocapsid protein in PBS buffer, 50 µL/well for 3 h at room temperature, or overnight at 4°C. Wells were washed using a plate washer (Zoom, Berthold Technologies, Germany) two cycles of three times with 100 µL of PBS/Tween 20 0.1%. Sera were diluted 200 times in PBS, nonfat milk 1%, and Tween 20 0.1%. Note that 50 µL of serum dilutions were incubated for 1 h at room temperature in their respective wells. Wells were washed two cycles of three times with 100 µL of PBS/Tween 20 0.1%. The Anti-Fc IgG VHH (Fc1) was derived from an antibody from immunized alpaca and expressed as a tandem with an optimized catalytic domain nanoKAZ from Oplophorous gracilirostris luciferase. Purified Fc1-nanoKAZ 1 ng/mL (400 × 106 RLU·s–1·mL–1) in PBS, nonfat milk 1%, and Tween 20 0.1% was loaded (50 µL/well) and incubated for 30 min at room temperature. Wells were washed two cycles of three times with 100 µL of PBS/Tween 20 0.1% then 50 µL of the luciferin solution was added (Promega).

Photons production was counted during 0.5 s per well and measured two times in a plate luminometer (Mithras2; Berthold, Wildbad, Germany).

Supplementary results

Table S1. Characteristics of participants of whom serum samples were obtained to correlate relative antibody units and neutralization activity from the virus neutralization studies.

Characteristic	Sub-group	N	%
Sex	Female	90	45
	Male	108	54
	Missing	106	
Age (median, range)		56	42 - 60
Immunity status	Infection-acquired	106	30
	AstraZeneca – one dose	11	4
	AstraZeneca – two doses	20	7
	Jansen – one dose	15	5
	Pfizer – two doses	89	29
	Sputnik – two doses	34	11

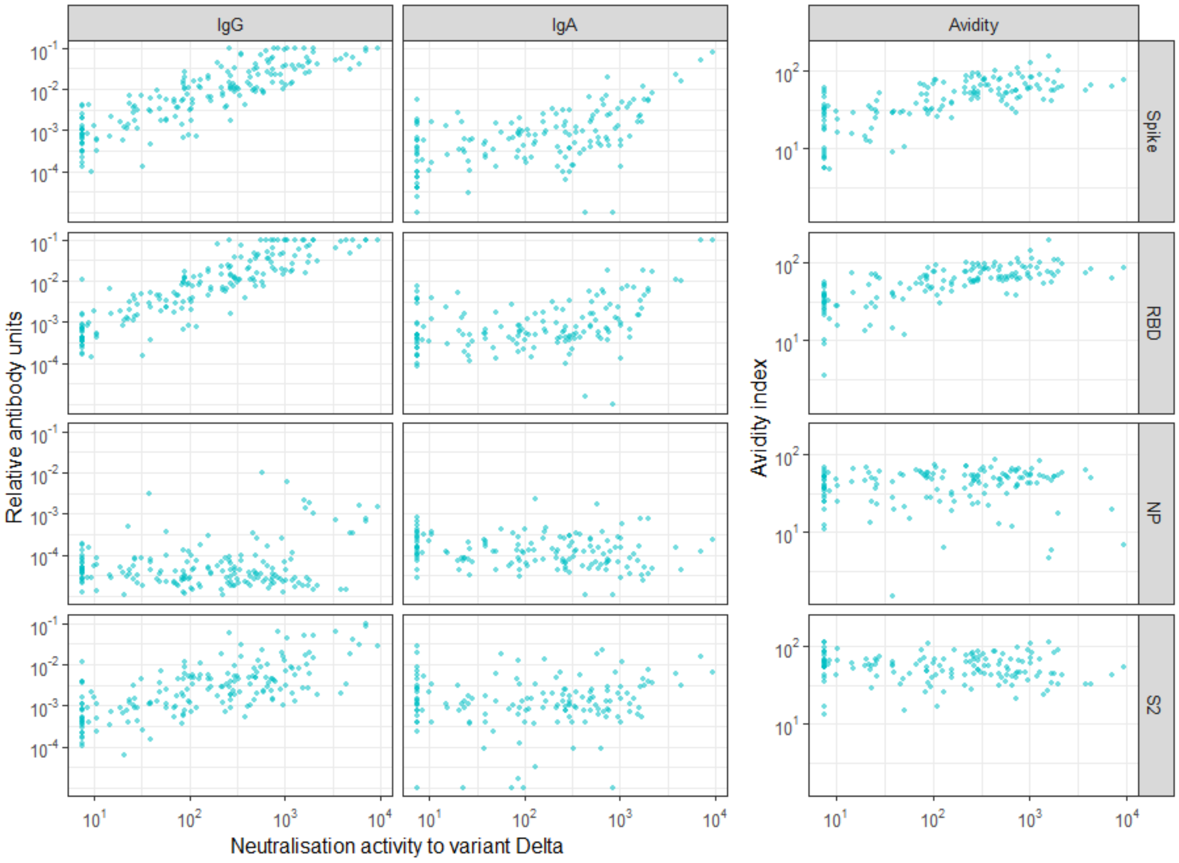

Figure S1. Correlation between relative antibody units and avidity index of four antigens with neutralization activity to variant Delta.

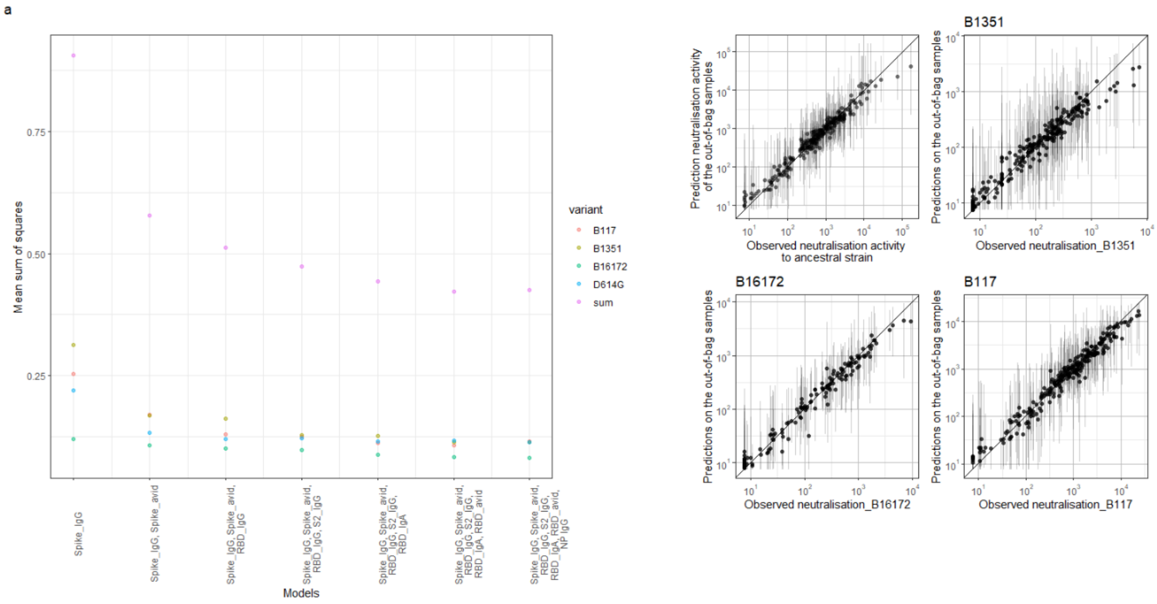

Figure S2. Development of a random forest regression model predicting neutralizing activity. (a) Basic regression model contained Spike IgG only. This model was complemented with the antigen producing the largest drop in the mean sum of squares for four models predicting neutralising activity to four strains. After model 6, no further reduction was observed. (b) These are the four random forest regression models predicting neutralising activity to D614G, B1351, B16172, and B117. The points reflect the means of the prediction of the out-of-bag samples. The vertical lines show the 2.5 and 97.5 percentile of the predictions.

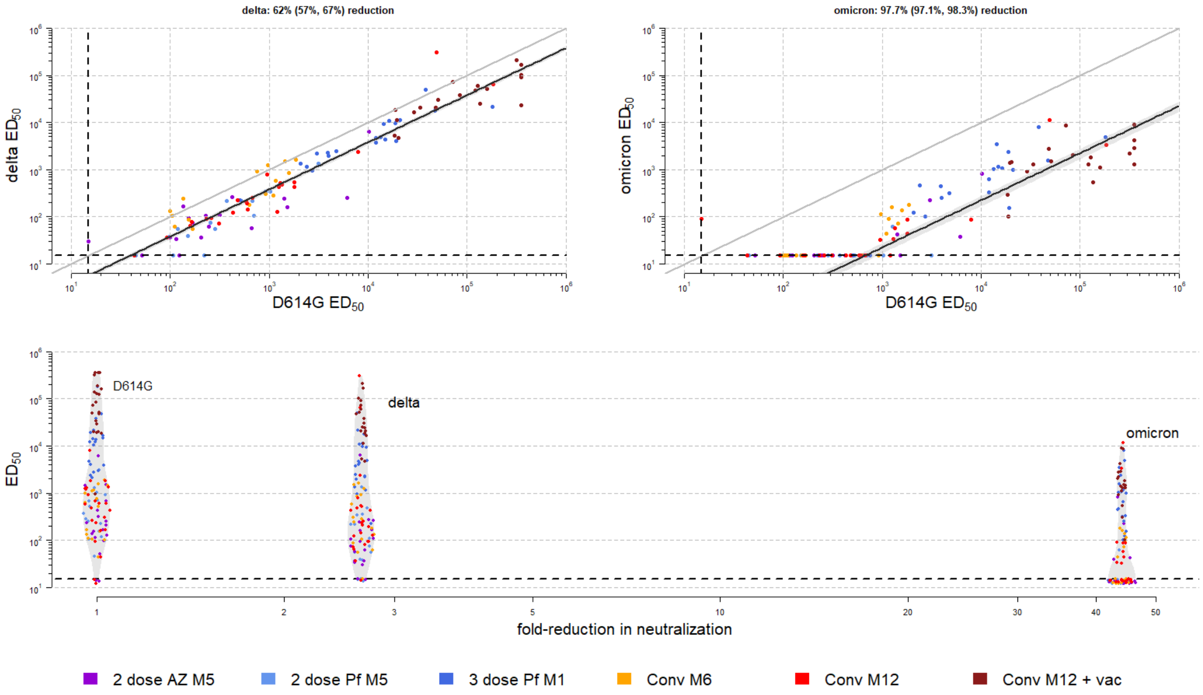

Figure S3. **Neutralization activity decreased almost 3-fold between ancestral strain and Delta, and more than 40-fold between ancestral variant and Omicron.** In two panels at the top, the fit of the censored linear regression is shown. In the figure in the lowest panel, this is converted into a fo*ld-reduction in neutralisation.*

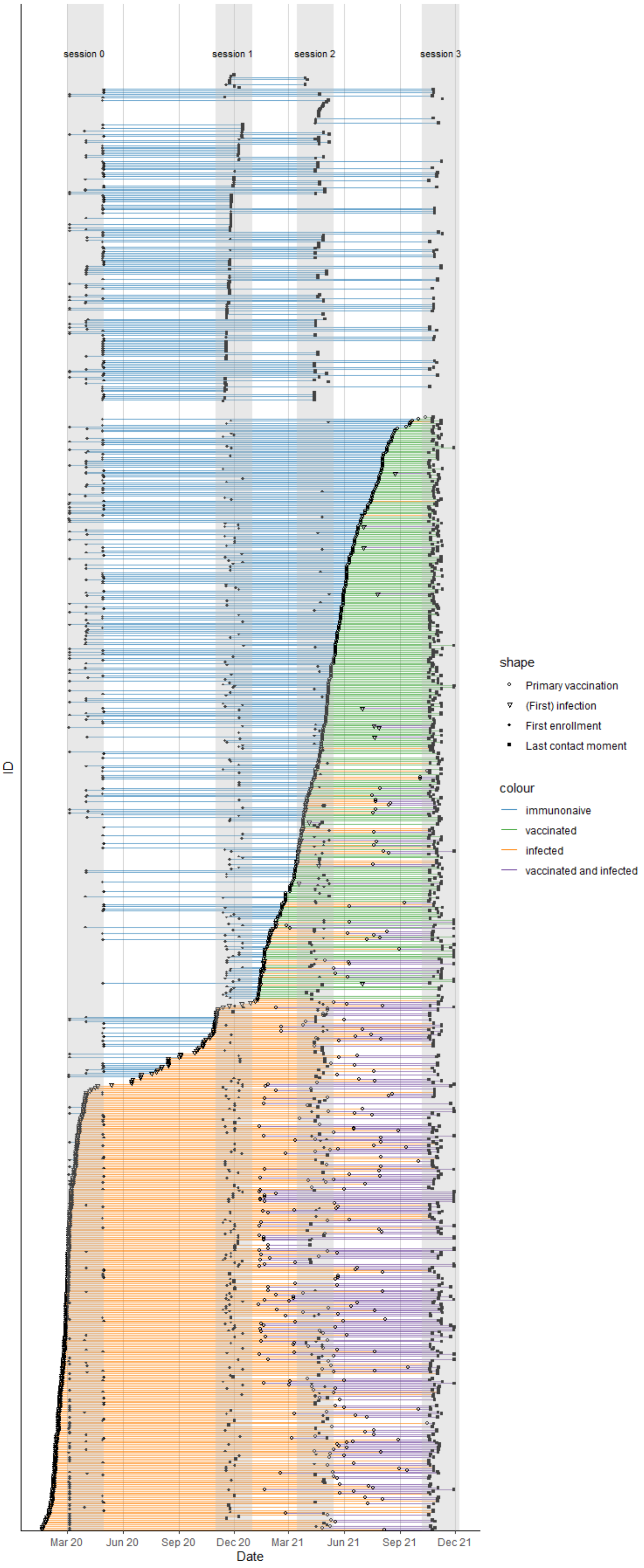

Figure S4. ***A complete visualization of all events in our study, including infections, vaccinations and enrolment activity in the longitudinal cohort study.*** *Colors depict the status of the participants in the study, which included immunonaive, vaccinated, infected, and infected and vaccinated.*

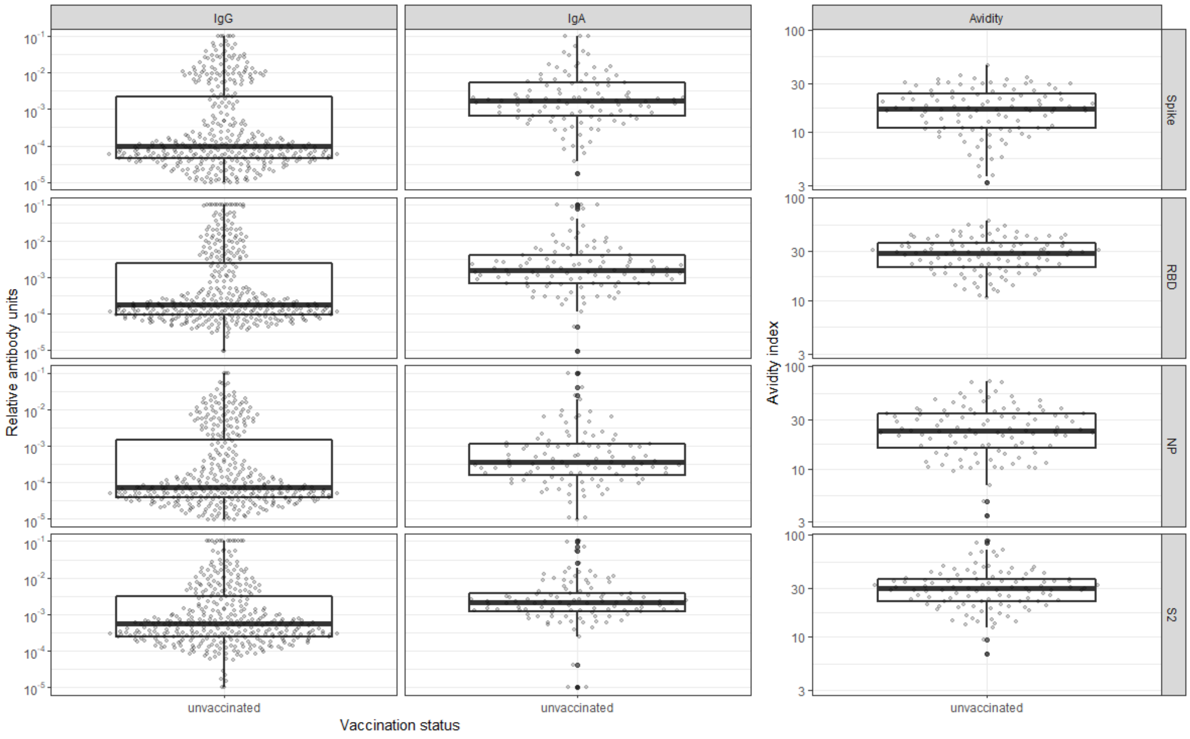

Figure S5. ***Antibody distributions by vaccination status, isotype and antigen in a cohort sampled around April 2020.***

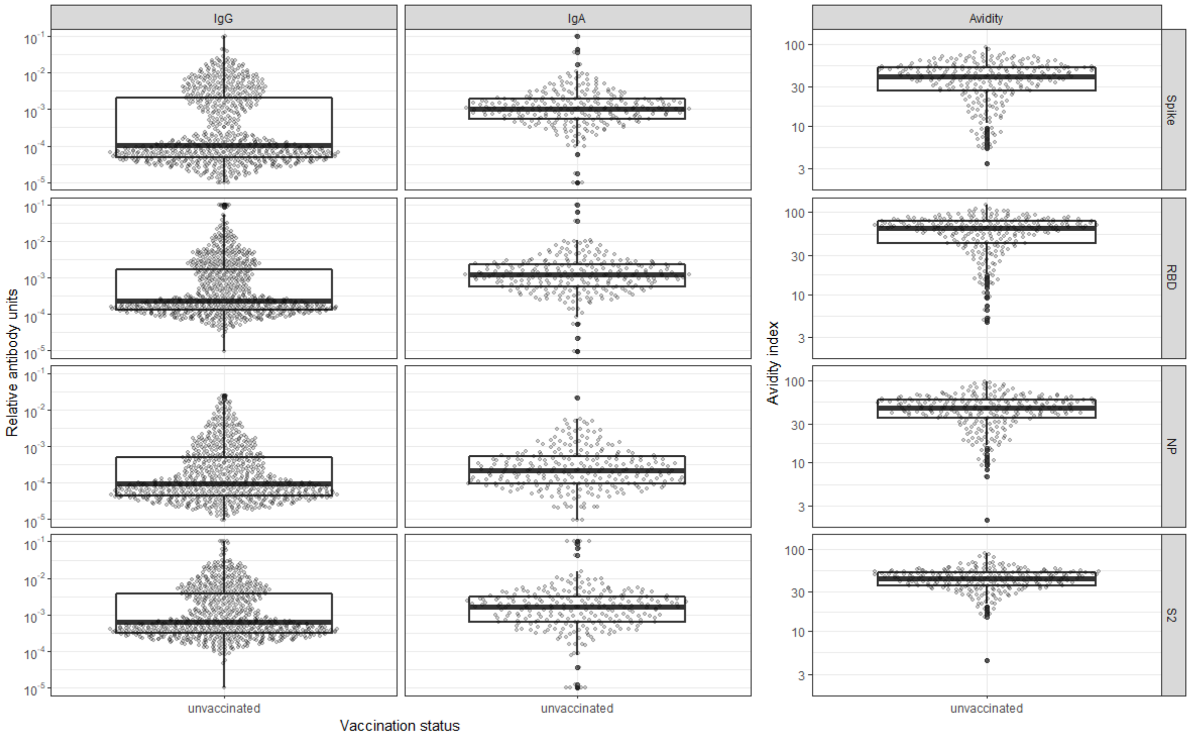

Figure S6. ***Antibody distributions by vaccination status, isotype and antigen in a cohort sampled around November 2020***

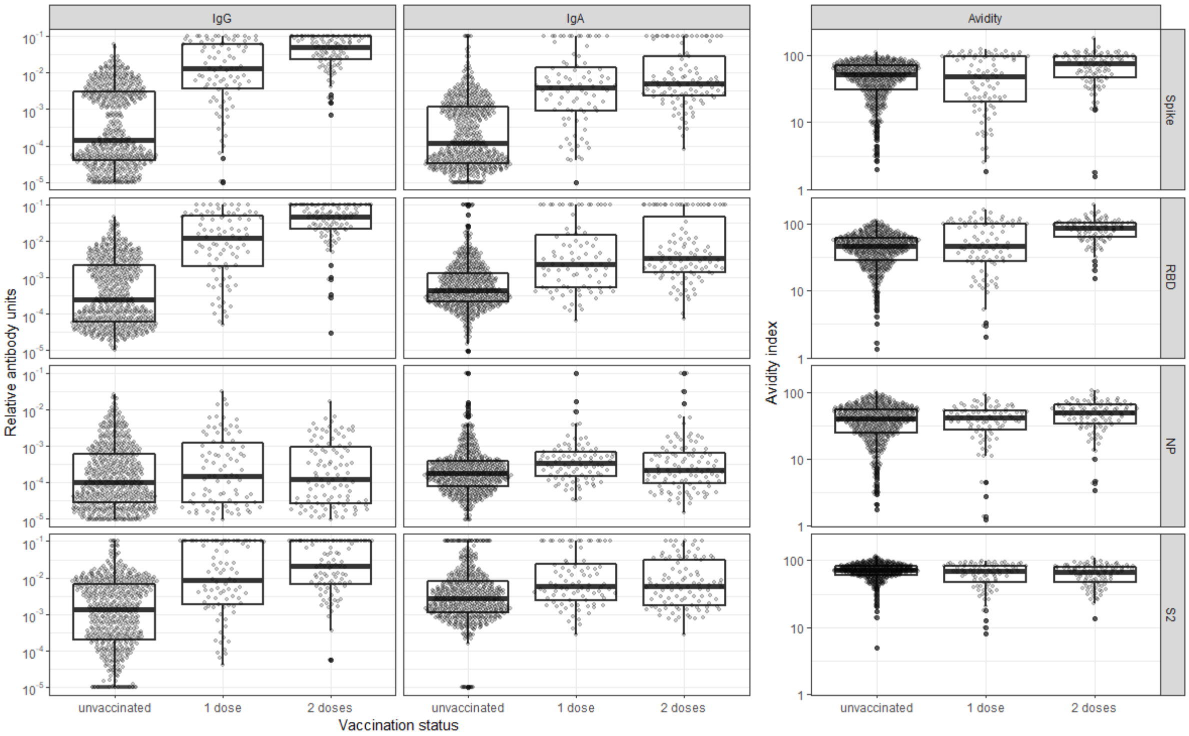

Figure S7. ***Antibody distributions by vaccination status, isotype and antigen in a cohort sampled around April 2021***

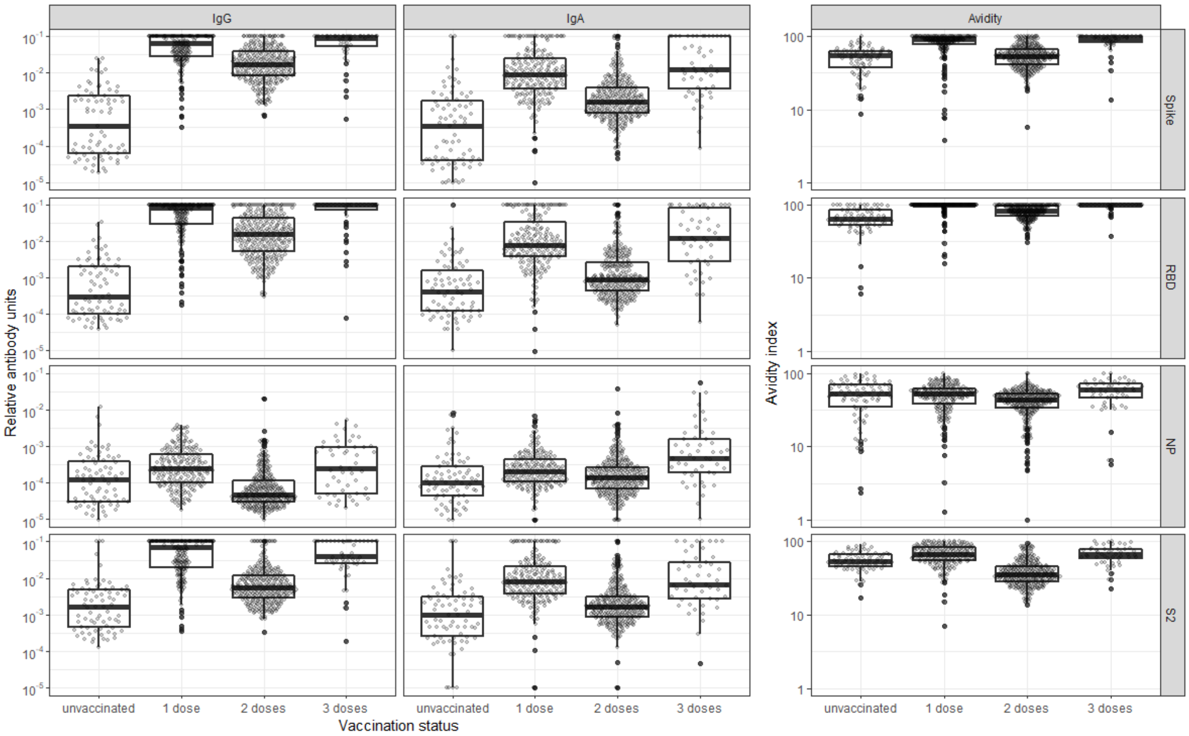

Figure S8. ***Antibody distributions by vaccination status, isotype and antigen in a cohort sampled November and December 2021.***

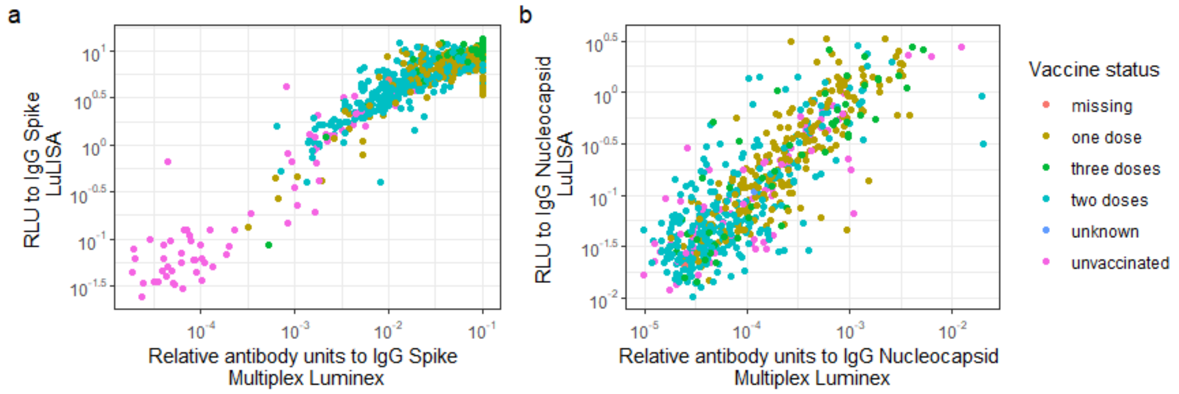

Figure S9. ***Measurements of antibodies to Spike (a) and Nucleocapsid (b) correlated well between the multiplex Luminex assay and the LuLISA assay.*** *A clear separation was observed between vaccinated and unvaccinated individuals. The samples shown here are from the COVID-Oise cohort in sampled in November 2021.*

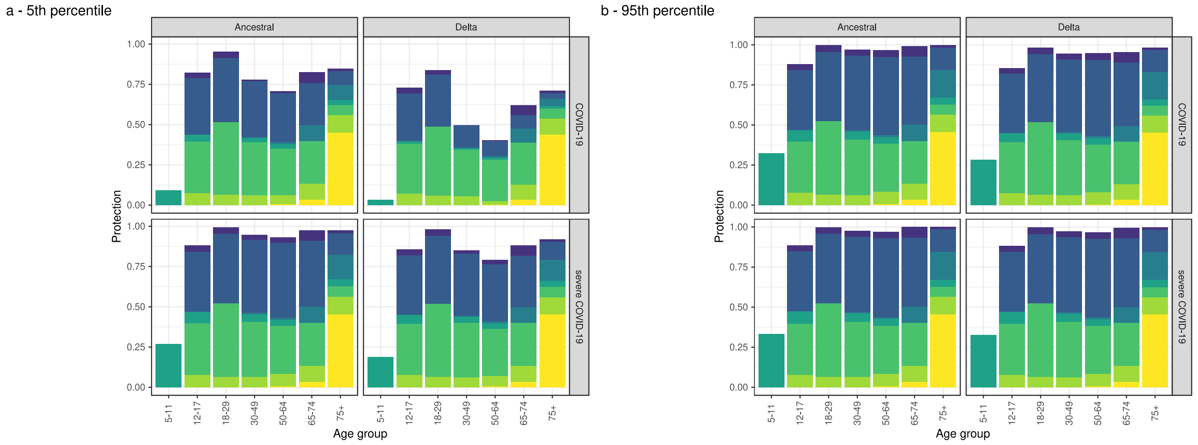

Figure S10. Aggregated protection against COVID-19 and severe COVID-19 in December 2021 of the longitudinal cohort. This figure is a complement visualizing the uncertainty of Figure 6 in the manuscript. (a) Stacked 5th percentile of reduced risks within each group to COVID-19 and severe COVID-19 by variant and age group. (b) Stacked 95th percentile of reduced risks within each group to COVID-19 and severe COVID-19 by variant and age group.

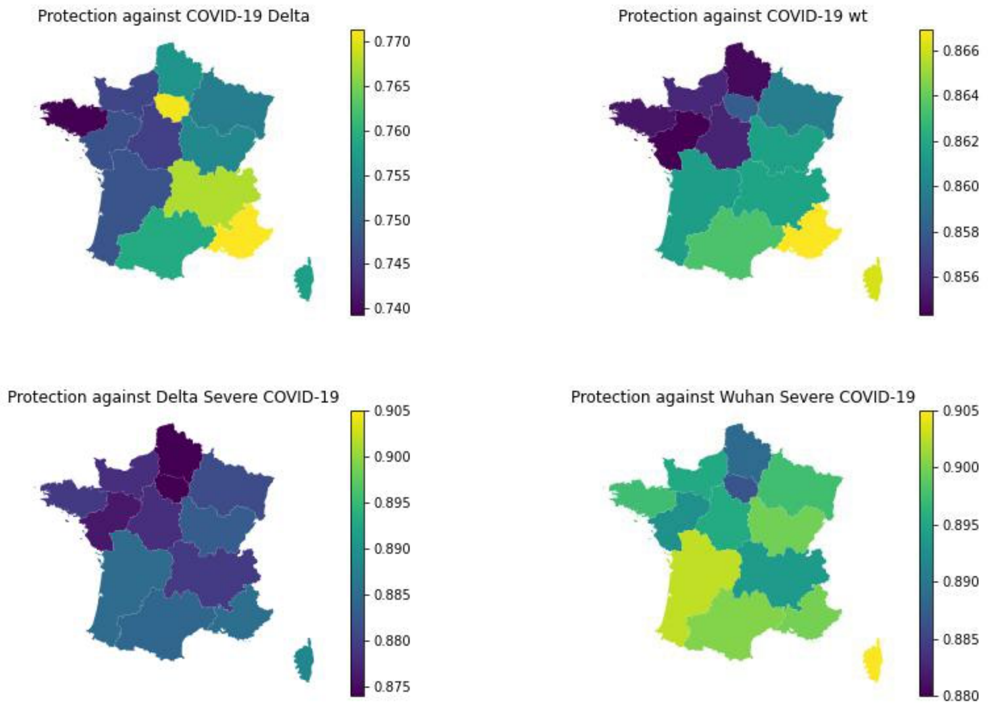

Figure S11. Protection estimates to COVID-19 and severe COVID-19 caused by the ancestral variant or Delta variant, by region. Protection estimates are aggregated estimates by age and dependent on immunity status. Vaccination status by age group were derived from Santé Publique France and infection status is derived in a similar manner as stated in Hozé et al (5).

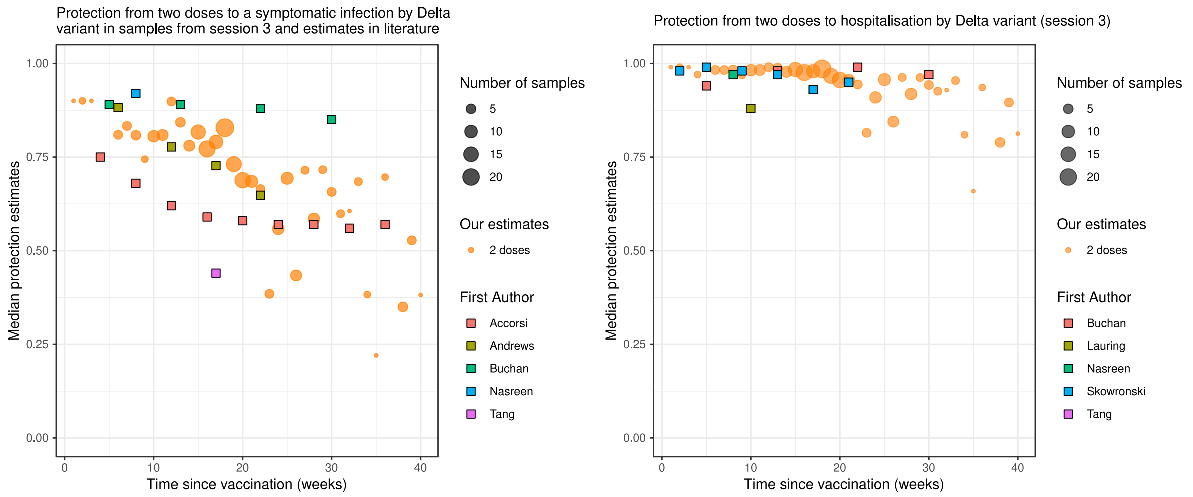

Figure S12. Estimated protection estimates and vaccine effectiveness estimates from literature correlate well. Our estimates by time since vaccination overlap with vaccine effectiveness estimates from the field. Vaccine effectiveness estimates are from (6-11).

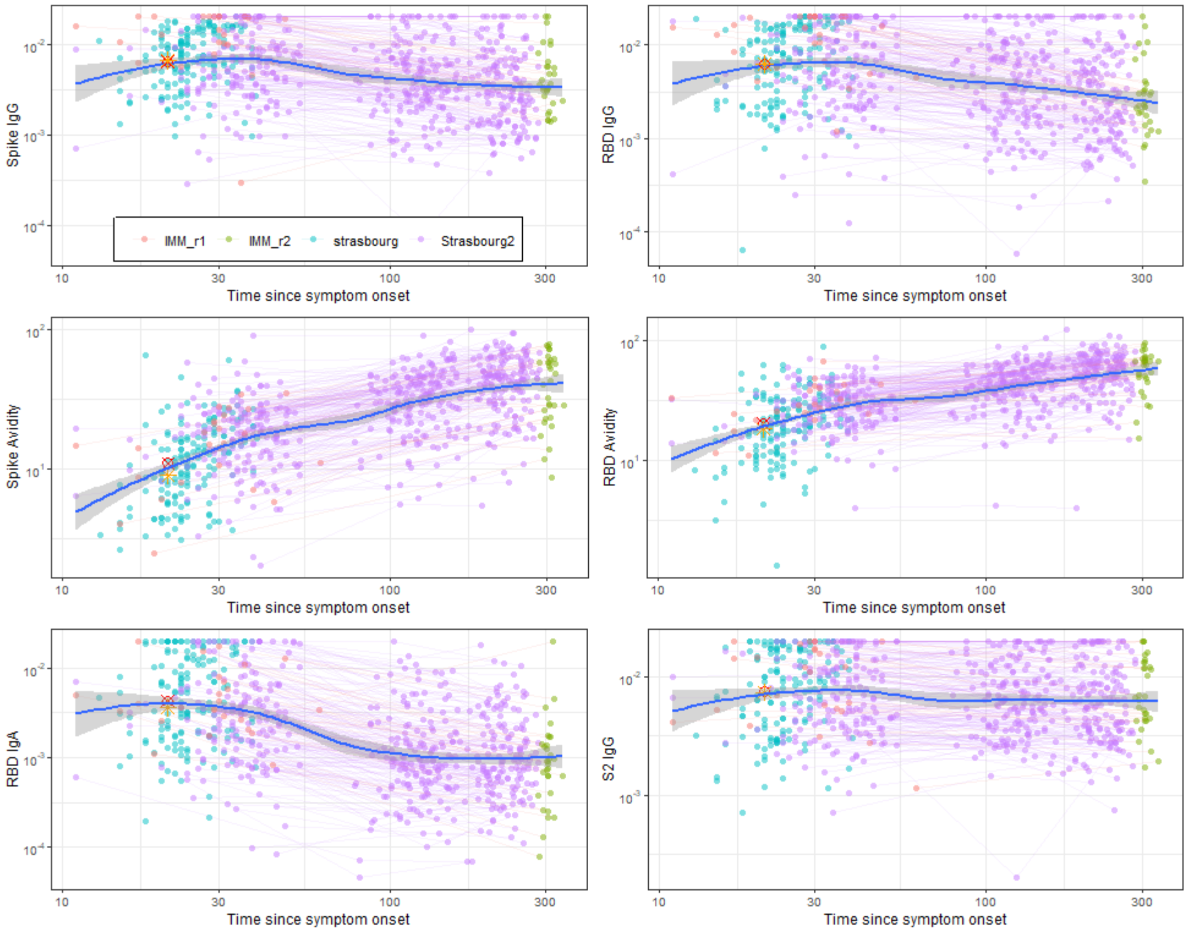

Figure S13. ***Estimation of convalescent serum.***

References

1. Pelleau S, Woudenberg T, Rosado J, Donnadieu F, Garcia L, Obadia T, et al. Kinetics of the SARS-CoV-2 antibody response and serological estimation of time since infection. The Journal of infectious diseases. 2021.

2. Hsieh CL, Goldsmith JA, Schaub JM, DiVenere AM, Kuo HC, Javanmardi K, et al. Structure-based Design of Prefusion-stabilized SARS-CoV-2 Spikes. bioRxiv : the preprint server for biology. 2020.

3. Buchrieser J, Dufloo J, Hubert M, Monel B, Planas D, Rajah MM, et al. Syncytia formation by SARS-CoV-2-infected cells. The EMBO journal. 2020;39(23):e106267.

4. Planas D, Bruel T, Grzelak L, Guivel-Benhassine F, Staropoli I, Porrot F, et al. Sensitivity of infectious SARS-CoV-2 B. 1.1. 7 and B. 1.351 variants to neutralizing antibodies. Nature medicine. 2021;27(5):917-24.

5. Hozé N, Paireau J, Lapidus N, Kiem CT, Salje H, Severi G, et al. Monitoring the proportion of the population infected by SARS-CoV-2 using age-stratified hospitalisation and serological data: a modelling study. The Lancet Public Health. 2021;6(6):e408-e15.

6. Nasreen S, Chung H, He S, Brown KA, Gubbay JB, Buchan SA, et al. Effectiveness of COVID-19 vaccines against symptomatic SARS-CoV-2 infection and severe outcomes with variants of concern in Ontario. Nature Microbiology. 2022;7(3):379-85.
7. Skowronski DM, Setayeshgar S, Febriani Y, Ouakki M, Zou M, Talbot D, et al. Two-dose SARS-CoV-2 vaccine effectiveness with mixed schedules and extended dosing intervals: test-negative design studies from British Columbia and Quebec, Canada. MedRxiv. 2021.
8. Tang P, Hasan MR, Chemaitelly H, Yassine HM, Benslimane FM, Al Khatib HA, et al. BNT162b2 and mRNA-1273 COVID-19 vaccine effectiveness against the SARS-CoV-2 Delta variant in Qatar. Nature medicine. 2021;27(12):2136-43.
9. Andrews N, Stowe J, Kirsebom F, Toffa S, Sachdeva R, Gower C, et al. Effectiveness of COVID-19 booster vaccines against covid-19 related symptoms, hospitalisation and death in England. Nature medicine. 2022:1-.
10. Buchan SA, Chung H, Brown KA, Austin PC, Fell DB, Gubbay J, et al. Effectiveness of COVID-19 vaccines against Omicron or Delta infection. medRxiv. 2022:2021.12. 30.21268565.
11. Lauring AS, Tenforde MW, Chappell JD, Gaglani M, Ginde AA, McNeal T, et al. Clinical Severity and mRNA Vaccine Effectiveness for Omicron, Delta, and Alpha SARS-CoV-2 Variants in the United States: A Prospective Observational Study. medRxiv. 2022.
